# From Donor to Patient: Collection, Preparation and Cryopreservation of Fecal Samples for Fecal Microbiota Transplantation

**DOI:** 10.3390/diseases8020009

**Published:** 2020-04-15

**Authors:** Carole Nicco, Armelle Paule, Peter Konturek, Marvin Edeas

**Affiliations:** 1 Cochin Institute, INSERM U1016, University Paris Descartes, Development, Reproduction and Cancer, Cochin Hospital, 75014 Paris, France; carole.nicco@inserm.fr; 2 International Society of Microbiota, 75002 Paris, France; armelle.paule@microbiota-site.com; 3 Teaching Hospital of the University of Jena, Thuringia-Clinic Saalfeld, 07318 Saalfeld, Germany; konturek@thueringen-kliniken.de

**Keywords:** fecal microbiota transplantation, FMT, cryoconservation, gut microbiota, fecal samples, stool banks, COVID-19

## Abstract

Fecal Microbiota Transplantation (FMT) is suggested as an efficacious therapeutic strategy for restoring intestinal microbial balance, and thus for treating disease associated with alteration of gut microbiota. FMT consists of the administration of fresh or frozen fecal microorganisms from a healthy donor into the intestinal tract of diseased patients. At this time, in according to healthcare authorities, FMT is mainly used to treat recurrent *Clostridium difficile*. Despite the existence of a few existing stool banks worldwide and many studies of the FMT, there is no standard method for producing material for FMT, and there are a multitude of factors that can vary between the institutions. The main constraints for the therapeutic uses of FMT are safety concerns and acceptability. Technical and logistical issues arise when establishing such a non-standardized treatment into clinical practice with safety and proper governance. In this context, our manuscript describes a process of donor safety screening for FMT compiling clinical and biological examinations, questionnaires and interviews of donors. The potential risk of transmission of SARS-CoV-2 virus by the use of fecal microbiota for transplantation must be taken urgently into consideration. We discuss a standardized procedure of collection, preparation and cryopreservation of fecal samples through to the administration of material to patients, and explore the risks and limits of this method of FMT. The future success of medicine employing microbiota transplantation will be tightly related to its modulation and manipulation to combat dysbiosis. To achieve this goal, standard and strict methods need to be established before performing any type of FMT.

## 1. Introduction

An imbalance of bacterial quantity and quality of gut microbiota has been linked to several pathologies. New strategies of microbiota manipulation have been developed such as Fecal Microbiota Transplantation (FMT) [1], the use of pre/probiotics, consumption of an appropriate diet, and phage therapy [2]. Fecal microbiota transplantation aims to replace pathogenic bacteria, a role usually performed by the use of antibiotics. Restoration of the microbial balance can be realized by over-populating the colon with commensal bacteria using fecal microbiota transferred from a healthy donor [3]. FMT is a procedure in which fecal matter, or stool, is collected from a tested donor, mixed with saline or another solution, strained, and either placed in a bank or directly into a patient, by colonoscopy, endoscopy, nasogastric tube, or enema. FMT is mainly used to treat recurrent *Clostridium difficile* [3,4]. This procedure could be also indicated to treat other diseases associated with alteration of gut microbiota [5].

The use of FMT was proposed from the 4th century AD and has been widely studied and documented since 2013, when the U.S. Food and Drug Administration approved FMT for the treatment of recurrent and refractory *Clostridium difficile* infections. In the 4th century yellow soup, as it was called in China, represented the feces used in patients with severe diarrhea. Accounts of treatment with fresh or fermented fecal suspensions applied in patients with gastrointestinal disorders, including diarrhea, constipation, and abdominal pain were described until the Chinese Ming Dynasty in the 16th century [6,7]. More recently, Eiseman and his colleagues successfully treated patients with FMT for Pseudomembranous colitis, in 1958, the first report in the medical literature [8]. With the increasing numbers of trials and the sharing of results around the world, mixed results have been observed, suggesting that heterogeneity in donor stool may play a role in patient response. Studies hypothesize that the microbiome is associated with a given indication [6,7,8]. Thus, fecal material collections should be informed by the health of the donors but also categorized to associate the donor with a recipient as part of a selection strategy. Ideally, information collected before the sampling should be used to identify which donors are efficacious. But our current knowledge is far from sufficient. The success of FMT in the treatment of *Clostridium difficile* infection (rCDI) has been demonstrated in several studies with a cure rates exceeding 90% [9,10,11]. According to the European consensus conference on fecal microbiota transplantation in clinical practice [12], FMT is recommended as a treatment option for both mild and severe rCDI. Infusion of fecal microbiota from a healthy donor into a recipient individual can restore the healthy microbial flora in the diseased colon, leading to the resolution of symptoms. In two open-label Randomized Controlled Trials (RCT) including patients with rCDI, FMT showed significantly higher resolution rates than the use of vancomycin (94% and 90% versus 31% and 26%, respectively) [9,12]. Moreover, in several systematic reviews and meta-analyses rCDI resolution rates achieved by FMT ranged between 85% and 89.7% [13,14,15]. In all these studies, FMT also showed an excellent safety profile, at least in the short-term follow-up, as only a few, mostly mild, adverse events were reported. Currently, long-term safety data are lacking and despite the increasing demand for FMT, rigorous exclusion criteria for donors strongly limit the widespread availability of suitable fecal material [16]. In theory, it may be possible to transmit potentially harmful microbiota traits, which may become not apparent for decades. One study showed that a drug resistant *E. coli* bacteremia has been transmitted by FMT [17]. However, this possibility should be taken into account in the context of a favorable risk-benefit ratio, as FMT is a highly efficient treatment for rCDI and can represent a life-saving treatment for affected patients. There are other possible applications of FMT in clinical practice, such as inflammatory bowel disease (IBD) [18], irritable bowel syndrome (IBS), antibiotic-resistant bacteria colonization, neuropsychiatric disorders, metabolic syndrome, and autoimmune diseases; but for none of these has an evidence-based recommendation to use FMT emerged. Moreover, several authors have shown that FMT can be effective in treating obesity in human and animals [19,20,21,22]. The lack of clarity about the exact mechanism of action and the ‘active ingredient’ of FMT (e.g., individual taxa or communities of bacteria, bacteriophages, or bioactive molecules such as bile acids) has hindered the ability to produce a standardized and well-characterized FMT product. FMT has an innate risk of transmittable infectious complications. It is not known whether a change of the microbiota following FMT has any long-term consequences. Despite a few worldwide existing stool banks, there is no standard method for producing material for FMT, and there are a multitude of factors that can vary between institutions offering this therapy [23]. To date, safety concerns and acceptability are the main constraints of therapeutic uses of FMT [24]. There are technical and logistical issues in establishing such a non-standardized treatment into clinical practice with safely, and with proper governance [25]. In view of this, an evidence-based recommendation is needed to drive the practical implementation of FMT in European countries. A careful donor screening covering fecal microbiota composition, pathogen status and undesirable antigens and ‘phenotypes’, must be preventively performed to create a bank of cryoconserved fecal microbiota [26,27,28].

## 2. Safety and Donor Screening

### 2.1. Clinical Examination, Questionnaire and Interview 

Donor selection is aimed to reduce and prevent adverse events related to the infused fecal material. All potential donors must submit to a personal interview concerning risk factors for transmittable diseases. The process begins with rigorous donor selection. Less than 3% of prospective donors qualify for the largest known Universal Stool Bank in the USA, and they must undergo a three-step rigorous process to be considered as an active donor [29]. Candidates undergo a pre-screen survey, and around 65% of individuals are excluded based on their responses, commonly for abnormal body mass index, logistical constraints and recent antimicrobial use. Participants who pass te pre-screening then undergo clinical assessment, with more than 80% participants excluded. The remaining candidates undergo laboratory investigations; and 50% of these candidates are rejected based on the presence of *Dientamoeba fragilis*, *Blastocystis hominis*, *Clostridium difficile*, and *Rotavirus* in stool samples [9,29,30]. Individuals aged 18 to 60 years are preferred. Exclusion criteria (Table 1 and Table 2) list the requirements of the European Commission for the selection of allogenic living donors of human tissue transplants (Commission Directive 2006/17/EC, 8 February 2006 [31] implementing Directive 2004/23/EC of the European Parliament and of the Council as regards certain technical requirements for the donation, procurement and testing of human tissues and cells).

The list of exclusion criteria will be probably extended in the future, depending on new findings concerning altered microbiota composition. In addition, local departments of infectious diseases and clinical microbiology should be consulted to adjust the screening to the local infectious pressure [32].

FMT can be obtained from related or unrelated donors. For some indications, the choice may be driven by specific needs. According to the available studies, there is no significant difference between relatives and unrelated donors [13,33], patient-selected and anonymous healthy donors in terms of FMT outcomes, at least as observed for rCDI [13]. The autologous fecal microbiota transplantation (Auto-FMT) is also another option. The auto-FMT procedure is well tolerated and effectively reestablished commensal bacterial populations while avoiding the potential introduction of viral or microbial agents that the patient had not previously encountered [34].

When the donors have been identified and selected by the clinician, stool samples can be taken, for a period of 28 days following consultation with the prescribing physician. Collection of stools must be conducted as described in Section 3.1. On this occasion, the patient is required to fill out a new questionnaire and the preparation will not be realized if the questionnaire highlights risky behavior (Table 2). The coronavirus disease 2019 (COVID-19) pandemic has affected more than one million persons worldwide and more than 50,000 deaths at the time of writing. The potential risk of transmission of SARS-CoV-2 virus from the use of fecal microbiota for transplantation is strongly highlighted by many health organisations.

Ianiro et al. from Italy, where the COVID-19 outbreak is spreading rapidly, stated that the national transplant centre has taken stronger measures and has recommended testing all potential tissue and stem-cell living donors, as well as dead donors, through real-time RT-PCR assays of nasopharyngeal swab samples (or bronchoalveolar lavage in deceased individuals) [35]. The European Society for Blood and Marrow Transplantation has recommended also excluding potential donors who have been diagnosed with COVID-19, and waiting at least 21 days before donation in those with a history of high-risk travel or contact [35]. On 23 March 2020, the US Food and Drug Administration (FDA) warned clinicians that SARS-CoV-2 virus may be transmitted in fecal samples during fecal microbiota transplants. The FDA recommends using stool donated before 1 December 2019, whenever possible. If stool donated before this date is not available, clinicians or researchers should screen donors for COVID-19, test donors or the stool for COVID-19, and develop informed consent forms that warn of potential transmission. 

Recent evidence from super donors in FMT must be taken into account and the concept of key phenotypes, species, and metabolites as predictors of FMT success must be anticipated. The possible effects of host genetics and diet on transplant and maintenance of FMT should also be considered. Therefore, banks must collect all useful information for the future. Further characterization of super donors will likely result in the development of more refined FMT formulations to help standardize treatment and reduce the variability of patient response [36]. A rationalization of the classification of donors in addition to their selection should optimize targeting and increase the probability of success of FMT clinical trials. A classification of genome- and microbiome-associated and metagenomic analysis (e.g., short-chain fatty acid concentration) of the host phenotype is a starting framework for rational donor classification by FMT banks [37]. The effectiveness of FMT probably depends on the ability of the donor to provide the necessary taxa capable of restoring metabolic deficits in recipients who contribute to the disease. The FMT banks have to be prepared to take into account the differences in donor stool efficacy FMT [38].

### 2.2. Biological Examination

Suitable donors for FMT should undergo both blood and stool testing no more than 4 weeks before donation. The primary purpose of donor testing is to investigate the presence of infectious diseases in the donor that are potentially transmittable to the recipient. The selected blood and stool exams allow an excellent safety profile in several Randomized Controlled Trial (RCTs) of FMT [9,11,12,39,40,41,42]. Some tests should be mandatory, associated with additional and optional tests, depending on geographical areas (e.g., human T-lymphotropic virus types I and II antibodies, or *Strongyloides stercoralis*), clinical conditions of recipients (e.g., cytomegalovirus IgG, viral capsid antigen IgG, bacterial culture for *Vibrio cholera* and *Listeria monocytogenes*, antigens and/or acid fast-staining for Isospora and Microsporidia in the case of immunosuppressed recipients) or assessment of the medical history of donors (e.g., calprotectin). Although for some potential pathogens (such as human T-lymphotropic virus) there is no knowledge on transmission via FMT, expanded screening tests can be realized in order to ensure a maximum safety profile for patients. Moreover, archiving of a sample of blood examined is required by the European Commission for the selection of allogenic living donors of human tissue transplants and by the French National Guidelines of FMT for rCDI [10,31,33,43,44,45,46,47,48,49]. Screening protocols for the detection of specific microorganisms in the intestinal tract differ between stool banks and evolve with time and new insights; therefore at least two aliquots of 50 mL must be kept for future biological analyses.

Two situations can be considered: (i) if all the results of the assessment are available and negative, then the FMT preparation can be validated by the pharmacist in charge. Or (ii) the screening 1 exam is not yet available. In the latter case, the preparation is frozen at −80 °C within 8 h after defecation to ensure the preservation and viability of the microbiota, and is stored in quarantine. 

Only when all the resulst of the questionnaire sheet are available, the pharmacist can commence the preparation of the fecal transplant, either for delivery directly to the recipient or for cryoconservation

## 3. Collection, Preparation and Storage of Fecal Material

### 3.1. Stool Collection 

If screening 1 is completed, stools should be collected from an individual donor within 1 month [33]. Screening has to be undertaken both before and after a period of donation to ensure that all stool collected and frozen between the two dates are safe [42]. Cleaning the equipment between donor stool processing is important to minimize the risk of cross contamination. Donors use a clean opaque plastic bag, which can be opened over a toilet to collect the stool and then sealed prior being placed in a larger storage bag. Donors have the option of donating on site or taking the bag home with a cooler box and ice pack so it can be delivered within 1 h of defecation. Stool can be stored for up to 8 h at 4 °C without significant impact on bacterial survival, but viability declines at room temperature or at 4 °C after more than 8 h [50].

### 3.2. Cryoconservation

There is no significant difference in the prevention of CDI relapse between patients randomized to frozen FMT versus fresh FMT. Robust evidence supports the practice of storing frozen stool preparations until they are ready to be administered [11]. The critical parameters that influence processing are temperature and time. Cryopreservation at temperatures ranging from −80 °C in electrical freezers to −196 °C in liquid nitrogen is a widely used method for storing bacteria [51]. Refreezing once defrosted is not permitted. Recently it has been shown that long-term preservation of transplanted feces at −20 °C can result in instability (especially concerning Actinobacteria and Bacteroidetes phyla) of the clinical outcome in FMT therapy [52].

Even though 30 g of fecal material were shown to be sufficient for successful FMT [39,53,54], the stool weight is a highly imperfect measure of microbiota quantity. There is wide variability in the microbial content in the stool samples between individuals and even between different donations. Thus approximately 60 g of stool are recommended for each treatment [55]. Fresh stool (25%) should be homogenized using sterile mortar and pestle with normal saline solution 0.9% (65%). The slurry is then successively passed through 2.0, 1.0, 0.5, and 0.25 mm stainless steel laboratory sieves (WS Tyler, Mentor, Oh) to remove undigested food and smaller particulate material. The resulting material passing through the 0.25 mm filter is centrifuged at 6000× *g* for 15 min in a Sorvall SS-34 rotor and resuspended to one-half the original volume in a non-bacteriostatic normal saline saline. The resulting concentrated fecal bacteria suspension is amended with sterile pharmaceutical grade glycerol (Sigma, St Louis, MO) as a cryoprotectant, to a final concentration of 10%. Once mixing is complete, the stool mixture is aliquoted into individual cryotolerant identified pots and immediately frozen at −80 °C [56]. The 250 mL pots used must be filled with no more than 200 mL of stool suspension as the liquid expands on freezing [53]. At least two aliquots of 50 mL must be kept for future biological analyses. During the month after collection another blood and stool analysis on the last samples must be conducted before the FMT (screening 2). Meanwhile, samples are escrowed for an additional 4 weeks in quarantine, to allow retesting of donors for HIV and hepatitis prior use of the inoculum store.

**Figure 1:**
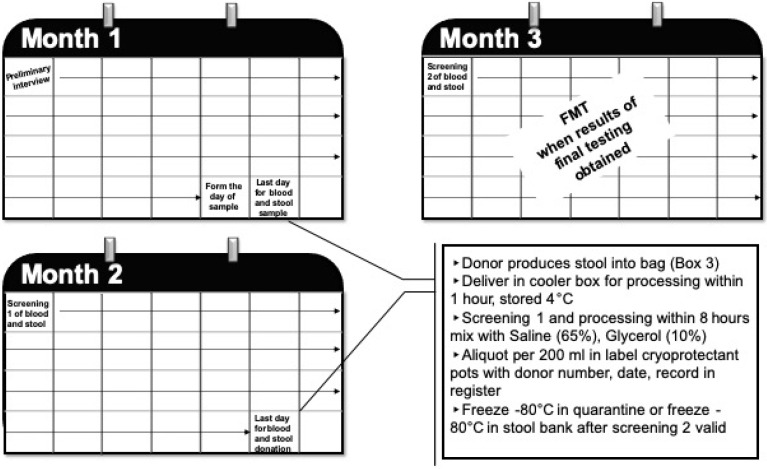
Maximal timing of the various mandatory steps to obtain a bank of ready-to-use FMT samples. The deadlines for carrying out the analyses after the samples can be reduced but are at the maximum of 1 month after the donation.

The European Tissues and Cells Directive (EUTCD) requires an expiration date to be defined and indicated in the Single European Code (SEC) when storing the material [57], but no studies have specifically examined microbial durability over time at −80 °C and −196 °C. In the European Union, human tissues and cells intended for human application need to be traceable from the donor to the recipient and vice versa (Figure 1).

**Figure 2:**
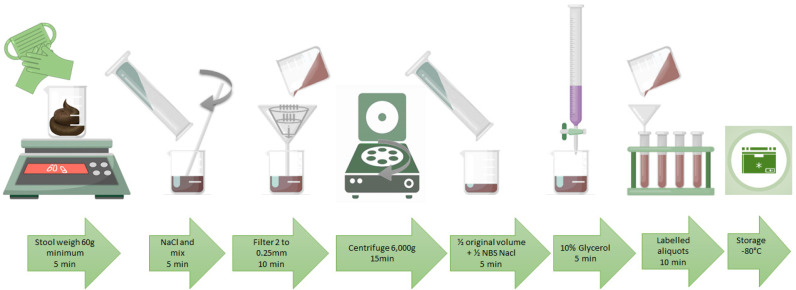
Schematic general steps for the preparation of fresh and frozen fecal material.

In this context, a unique identifier, called the Single European Code (SEC) has been introduced [58]. The SEC is an alphanumeric code that carries information on the Tissue Establishment (TE), the donation number, the product code, divisions, and expiration date in a standard format. The identification number should be recorded in a secure donor document along with contact details and screening results, thus the donor is de-identified to the recipient but can be traced in the event of illness developing in the recipient. The general steps (Figure 2 and Table 3) recommended by this statement are based on what has been described, but never rigorously tested. There are no studies reported comparing different preparation protocols of fresh fecal material, but the protocols used in different studies are comparable and allow good/moderate evidence of suitable protocols of fresh fecal preparation for FMT treatments of rCDI. Both anaerobically and aerobically prepared samples are efficient in the treatment of rCDI [9,39,44,53,54].

There are several in-vitro and clinical studies supporting the stability of material for FMT when stored in these conditions [11,59]. Banked material is stored for a maximum of six months before being discarded as clinical waste even though it could theoretically be kept longer (Table 4). Refreezing once defrosted is not permitted.

### 3.3. Sample Testing

General laboratory screening tests (made within 30 days of donations) are summarized in Table 5. All results values have to be within normal range regardless age and sex. Other tests, such as PCR or Western blots can be performed to evaluate the quality of the microbiota contained in the stool sample. A large international consortium of researchers compared the effects of numerous technical approaches in every single step of the gut microbiota analysis in several independent laboratories [59,60]. This study identified differences in the DNA extraction method as the biggest influence on the downstream gut microbiota analysis results. This analysis needs to be standardized and therefore the DNA extraction also. A protocol named ‘Protocol Q’ was identified as the best option and was proposed as a benchmark for the DNA extraction from human feces [59]. Concentration and quality of the obtained fecal DNA can be measured by NanoDrop analysis, in which the absorbance ratios at 260 nm/230 nm and 260 nm/280 nm can be determined to evaluate the purity of the extracted DNA, which should be around 2 and 1.8, respectively. Downstream phylogenetic 16S rDNA microbiota analysis starts with a PCR on 25 ng of fecal DNA. Therefore, since this protocol generates at least 1 μg of DNA at a concentration of 5 ng/μL, the quantity of obtained DNA is not a limiting factor. The procedures for phylogenetic 16S rDNA microbiota analyses have been previously outlined [49]. The sequencing of the various steps to obtain a bank of ready-to-use FMT samples is summarized in Figure 2.

## 4. Administration of Fresh and Frozen FMT

Among patients with recurrent or refractory CDI, frozen FMT is as effective as fresh FMT in clinical resolution of diarrhea [49,54,61]. In consideration of the potential advantages of performing frozen FMT, it is a reasonable option to select frozen FMT. The administration to the recipient must be performed under medical supervision, after signing informed consent in the context of hospitalization (possible outpatient).

In the case of immediate administration, the stools are diluted in sterile physiological saline (without glycerol), homogenized, filtered as previously described and administered within less than 6 h after the emission. For frozen FMT, thawing is done over 2–4 h in an ice bath before the FMT procedure [62]. The current routes of administration of fecal material vary according to the teams and pathologies. The administration can be performed through upper GI route (via esophagogastroduodenoscopy (EGD), nasogastric (NGT), nasojejunal, or nasoduodenal tube), lower GI route (via colonoscopy or retention enema), and oral capsule. In general, FMT via the upper GI route is realized in patients with an inflamed colon. However, the discomfort sensation during tube placement, risks of aspiration, and inability to evaluate the colon mucosa or to collect mucosa tissue samples are weak points. The use of NGT provides more important exposure of donor fecal bacterial flora in the digestive tract of recipients. It allows transplanted flora a high likelihood of sufficient survival, and results in overcoming the possible growth of pathogens. NGT is well tolerated and exhibited fewer significant insertion-related complications than colonoscopy [63]. FMT via colonoscopy has superiority in recolonizing the entire colon with favorable bacteria. Thus, colonoscopy-guided FMT allows the physician to directly evaluate the severity of inflammation, and select preferential sites for application of a sufficient amount of feces [44]. Additionally, the incorporated bowel lavage can reduce the existing pathogenic content and facilitate colonization of healthy donor microbiota [9]. Colonic lavage before FMT may not contribute to clinical resolution of diarrhea [64]. FMT via retention enema is more affordable and less invasive than colonoscopy, but the donor fecal material cannot be delivered to the entire colon, thus this method is limited to the distal colon [65,66,67]. Oral capsules for FMT administration has several advantages including, less invasiveness and higher patient acceptability, but the expense and large capsule burden are disadvantages [14,25]. Studies suggest that lower gastrointestinal FMT delivery may be preferable compared to upper gastrointestinal FMT delivery [63,68]. The use of capsule or enema is less invasive, does not require specialized, costly devices, and can be performed outside a care facility [11,42]. However, there is no widespread consensus on the optimal FMT route of administration. In all cases, an immediate medical follow-up of the recipient and prolonged follow-up of the recipient and the donor are realized.

## 5. Limits and Risks

On the basis of currently available clinical studies, FMT is considered as a validated therapeutic alternative for the management of recurrence of *Clostridium difficile* infections. Conversely, if other pathologies such as inflammatory bowel diseases, intestinal functional disorders, obesity, metabolic and autoimmune diseases, seem today to represent potential targets for the use of this therapy, the level of evidence currently is not sufficient to recommend this practice routinely yet. The practice of FMT outside the management of recurrences of CDI is therefore a matter for clinical research. Until now, adverse effects observed in the short-term in immunocompetent patients following a FMT, remain minor. They mainly include diarrhea, constipation, bloating or even abdominal discomfort, and low-grade fever appearing in the hours following transplantation and for never more than 48 h. Some case of deaths have been recorded [10,17,66,67]. However, these may be attributable to the recurrence of the underlying CDI, a gastrostomy tube leak or colonic distension but not the FMT. No other directly attributable long-term adverse effect of FMT in over 600 cases in the literature has been observed [68]. There is a lack of long-term study data [69] and thus the eventual long-term risk needs to be taken into consideration in any screening protocol and discussed with patients. No cases of sepsis or other infections have been reported. However, we must add, to these effects directly attributable to transplantation, those related to the mode of administration when it is done by nasogastric tube or by colonoscopy. Every recipient of FMT needs to be informed about the potential risks before the procedure.

The possible long-term complications are not known but they may appear if this therapy develops. Therefore, the mandatory steps described must be carefully followed. The EUTCD requires safety procedures and notification systems to be in place in case of any serious adverse events Commission Directive (EU) 2015/565 [57]. In order to monitor and reduce these events, specific requirements for traceability and a community procedure for notifying the serious adverse reactions and events, are listed in annex III and IV of the Commission Directive (EU) 2015/565 [57] of 8 April 2015 amending Directive 2006/86/EC. In case of any safety procedures Adverse (AE) and Serious Adverse Events (SAE) related to procedural handling of the feces in the laboratory, the probable causes must be determined, and new correcting procedures should be implemented that describe how to handle these problems. For example, safety samples can be collected from thawed and used suspensions to ensure full traceability. These safety samples could be stored in a −80 °C freezer for 30 years, ready to thaw and screen in case of AE/SAE to investigate the potential contributing causes.

Finally, to maintain good laboratory practices, only specially trained staff can perform the tasks. In concordance with EUTCD, training procedures are performed every year and kept up to date in case of updates [70]. It must be underscored that additional support on training of tissue establishments personnel was given through EU-funded projects, such as European Quality System for Tissue Banking (EQSTB) [71] and European Good Tissue Practices (EuroGTPs, http://goodtissuepractices.eu/). The good practices on quality management, responsible persons and personnel developed by the EU-funded initiatives were also included by the Council of Europe in a dedicated guide to the quality and safety of tissues and cells.

## 6. Conclusions

The FMT is an effective treatment option, which is now widely recognized. The FM corresponds to the definition of a drug whose preparation is submitted to essential rules of hygiene, safety and traceability. With its increased use in a clinical setting, regulation and standardization become urgent. During our last clinical study [5], we compared the different methods of collection, preparation and cryoconservation of fecal gut microbiota from hemodialysis patients. This study will allow us to perform FMT in these patients in the best conditions. This manuscript describes an example of FMT processes to follow across the different steps from the donor to the administration to the patient, including donor screening, collection, preparation and cryopreservation of fecal material. The impacts of coronavirus disease on our health system and society are huge. The potential risk of transmission of SARS-CoV-2 virus by the use of fecal microbiota for transplantation must be taken in consideration. Every donor has to be checked for the presence of SARS-CoV-2 even if symptoms are not present, because this infection can be present in many individuals without symptoms [35].

Objectives of this manuscript were to provide a potential standardization in the context of bank stool creation and to highlight risks and limits of the FMT procedure. The future of medicine involving microbiota will be tightly linked to microbiome modulation, manipulation and equilibrium. To achieve this goal, we need standard and strict methods for collection, preparation and cryopreservation of fecal material before performing any type of FMT. 

The other strategic point which must be taken into consideration for microbiota medicine is the digital revolution, which will transform the way we prevent and treat many diseases. We need to be ready to provide the most elaborate microbiota signature for each individual. We also have to conserve fecal material early in life, and digitize their characteristics and place of storage, to be ready for the next revolution of artificial intelligence.

## Figures and Tables

**Table 1:** List of key issues of the European Commission used to select potential donors at the preliminary interview.

1	Exposure to HIV 1-2, HBV or HCV, syphilis, human T-lymphotropic virus I and II, malaria, trypanosomiasis, tuberculosis
2	Systemic infection not controlled at the time of donation
3	Use of illegal drugs
4	Risky sexual behaviour (anonymous sexual contacts; sexual contacts with prostitutes, drug addicts, individuals with HIV, viral hepatitis, syphilis; work as prostitute; history of sexually transmittable disease)
5	Previous reception of tissue/organ transplant
6	Previous (<12 months) reception of blood products
7	Recent (<6 months) needle stick accident
8	Recent (<6 months) body tattoo, piercing, earring, acupuncture
9	Recent medical treatment in poorly hygienic conditions
10	Risk of transmission of diseases caused by prions
11	Recent parasitosis or infection from rotavirus, *Giardia lamblia* and other microbes with gastrointestinal (GI) involvement
12	Recent (<6 months) travel in endemic areas of gastrointestinal pathogens
13	Recent (<6 months) history of vaccination with a live attenuated virus, if there is a possible risk of transmission
14	Healthcare workers (to exclude the risk of transmission of multidrug-resistant organisms)
15	Individual working with animals (to exclude the risk of transmission of zoonotic infections)
16	Metabolic and neurological disorders
17	History of IBS, IBD, chronic constipation, coeliac disease, other chronic GI disorders
18	History of chronic, systemic autoimmune disorders with GI involvement
19	History of, or high risk for, GI cancer or polyposis
20	Recent appearance of diarrhea, hematochezia
21	History of neurological/neurodegenerative disorders
22	History of psychiatric conditions
23	Overweight and obesity (BMI > 25)
24	Drugs that can impair gut microbiota composition
25	Recent (<3 months) exposure to antibiotics, immune-suppressants, chemotherapy
26	Chronic therapy with proton pump inhibitors

**Table 2:** List of key issues to select confirm donors at the donation day interview.

1	Newly appeared gastrointestinal (GI) signs and symptoms, for example, diarrhea, nausea, vomiting, abdominal pain, and jaundice
2	Newly appeared illness or general signs as fever, throat pain, swollen lymph nodes
3	Use of antibiotics or other drugs that may impair the gut microbiota, new sexual partners or travels abroad since the last screening
4	Recent ingestion of a substance that may result harmful for the recipients
5	Travel in tropical areas, contact with human blood (sting, wound, showing, piercings, tattoos), and sexual high-risk behaviour
6	Diarrhea (more than three loose or liquid stools per day) among members of the entourage (including children) within 4 weeks’ prior to donation

**Table 3:** List of general steps for stool collection and the preparation of fresh and frozen fecal material.

Stool Collection
Use a sterile opaque plastic bag that can be opened over a toilet
Seal with a cable tie and placed in a larger clean ziplock bag
Donors have the option of donating on site or taking the bag home with a cooler box and an ice pack so it can be delivered within 1 h of defecation.
Stool can be stored for up to 8 h at 4 °C maximum
**Frozen Fecal Material**
A dedicated sterile hood, disinfected with measures that are effective against sporulation bacteria, should be used
The use protective gloves and facial masks during preparation
60 g of donor feces and 150 mL of saline solution should be used
The slurry is successively passed through 2.0, 1.0, 0.5, and 0.25 mm stainless steel sieves
The resulting material is centrifuged at 6000× *g* for 15 min
The pellet is resuspended to one-half the original volume in a non-bacteriostatic (NBS) normal saline
Before freezing, glycerol should be added up to a final concentration of 10%
Aliquot the final suspension into a sterile individual cryotolerant pots, clearly labelled and traceable
Stored at −80 °C
The day of fecal infusion, fecal suspension (Stool (25%), saline 0.9% (65%), glycerol (10%) should be thawed in a warm (37 °C) water bath and infused within 4 h from thawing
After thawing, saline solution can be added to obtain a desired suspension volume.
Aliquots are essential to avoid refreezing

**Table 4:** List of existing worldwide stool banks.

Place, Date of Creation	Legal Authorization	Donors	Products	Indications
Leiden University Medical Centre, The Netherlands, 2015	Allowed for CDI, no legal guideline	Healthy unrelated donors, unpaid	Fresh/frozen stool samples	Recurrent/refractory CDI Pilot study for IBS clinical trial for MDR bacteria
OpenBiome, Somerville, MA, USA, 2012	Regulated as an investigational biologic, ‘enforcement discretion’ permits use of FMT for rCDI without IND	Rigorously screened universal donors; compensated $40 per donation	Fresh/frozen stool samples in three delivery formats: upper delivery, lower delivery, and oral delivery (capsules)	CDI not responding to standard therapies Clinical trials for all other indications
PHE Public Health Laboratory Birmingham, UK, 2015	MHRA manufacturers’ licence needed for clinical trial use. Special licence for CDI	Healthy unrelated donors, unpaid	Fresh/frozen stool samples	Recurrent/refractory CDI
Portsmoth Hospitals, Portsmouth, UK, 2013	Officially under MHRA as a medicinal product	Healthy, unrelated donors, unpaid	Fresh/frozen stool samples (frozen since July 2015)	Recurrent/refractory CDI
Saint-Antoine Hospital, AP-HP, Paris, France 2014	Allowed for CDI (considered as a drug) Clinical trial for other indications	Healthy related or unrelated donors, unpaid (paid for clinical trial)	Fresh frozen stool samples	Recurrent CDI Clinical trial for Crohn’s disease
University Hospital Cologne, Germany, 2014	No legal guideline	Healthy, unrelated donors, unpaid	Frozen preparations for endoscopic application, enema or in capsules	Recurrent CDI
Hospital Ramo’n y Cajal, Madrid, Spain, 2016	No legal guideline	Healthy, unrelated donors, unpaid	Fresh frozen stool samples	Recurrent CDI, in principle local patients only
Medical University Graz, Austria, 2012	Allowed for CDI based on national guideline Other indications need ethics committee board approval	Healthy related and unrelated volunteers. Clinical trials compensated with V50/donation	Fresh and frozen faecal samples ready to use for lower GI endoscopy	Recurrent CDI; Severe CDI Idiopathic colitis; Colitis in critically ill; Clinical trials for UC, IBS, GvHD patients
Asia Microbiota Bank, Hong Kong, 2016	No legal guideline	Healthy, unrelated donors, unpaid	Frozen processed microbiota samples (no fresh or whole stool samples available clinically)	Recurrent CDI Primary CDI Clinical trial for IBS, IBD and MDR bacteria

**Table 5:** Blood and stool testing to check donors for any potentially transmittable disease (Screening 1).

	General Testing	Specific Testing
**Blood**	▸ Cytomegalovirus, Epstein-Barr virus, Hepatitis A, HBV, HCV, Hepatitis E, Syphilis, HIV-1 and HIV-2, *Entamoeba histolytica* ▸ Complete blood cell count with differential ▸ C-reactive protein and erythrocyte sedimentation rate ▸ Albumin ▸ Creatinine and electrolytes ▸ Aminotransferases, bilirubin, gamma-glutamyltransferase, alkaline phosphatase	▸ Human T-lymphotropic virus types I and II antibodies ▸ *Strongyloides stercoralis*
**Stool**	▸ Detection of *Clostridium difficile* ▸ Detection of enteric pathogens, including *Salmonella*, *Shigella* ▸ *Campylobacter*, *Escherichia coli* O157 H7, *Yersinia*, vancomycin-resistant enterococci, methicillin-resistant *Staphylococcus aureus*, Gram-negative multidrug-resistant bacteria ▸ *Norovirus* ▸ Antigens and/or acid fast staining for *Giardia lamblia* and *Criptosporidium parvum* ▸ Protozoa (including *Blastocystis hominis*) and helminths ▸ Fecal occult blood testing	▸ Detection of *Vibrio cholera* and Listeria monocytogenes ▸ Antigens and/or acid fast staining for Isospora and Microsporidia ▸ Calprotectin ▸ *Helicobacter pylori* fecal antigen ▸ Rotavirus
